# Comparison of the Inhibitory Binding Modes Between the Planar Fascaplysin and Its Nonplanar Tetrahydro-β-carboline Analogs in CDK4

**DOI:** 10.3389/fchem.2021.614154

**Published:** 2021-02-18

**Authors:** Yan Liang, Huili Quan, Tong Bu, Xuedong Li, Xingang Liu, Songsong Wang, Dian He, Qingzhong Jia, Yang Zhang

**Affiliations:** ^1^ Materia Medica Development Group, Institute of Medicinal Chemistry, Lanzhou University School of Pharmacy, Lanzhou, China; ^2^ The Fourth Hospital of Shijiazhuang, Shijiazhuang, China; ^3^ Department of Pharmacology, Hebei Medical University, Shijiazhuang, China; ^4^ The Second Hospital of Hebei Medical University, Shijiazhuang, China

**Keywords:** fascaplysin, nonplanar, MTT assay, molecular docking, MD simulation

## Abstract

Fascaplysin is a natural marine product originating from sponges, attracting widespread attention due to its potential inhibitory activities against CDK4. However, its clinical application has been largely limited because of serious adverse effects caused by planar skeleton. To reduce the serious adverse effects, 18 tetrahydro-β-carboline analogs (compounds 6a-i and 7a-i) were designed and synthesized via breaking the planarity of fascaplysin, and the biological activities of the synthesized compounds were evaluated by MTT assay and CDK4/CycD3 enzyme inhibition assay. The title compounds showed varying degrees of inhibitory activities, especially the cytotoxicity of compound 6c against HeLa cells (IC_50_ = 1.03 ± 0.19 μM) with quite weak cytotoxicity toward the normal cells WI-38 (IC_50_ = 311.51 ± 56.06 μM), and the kinase inhibition test indicated that compound 6c was a potential CDK4 inhibitor. In order to further compare the action mechanisms of planar and nonplanar molecules on CDK4, the studied complexes of CDK4 bound with fascaplysin and three representative compounds (compound 6a-c) with bioactivities gradient were constructed by molecular docking and further verified through molecular dynamic simulation, which identified the key residues contributing to the ligands’ binding. By comparing the binding modes of the constructed systems, it could be found that the residues contributing significantly to compound 6c′s binding were highly consistent with those contributing significantly to fascaplysin’s binding. Through the design, synthesis of the nonplanar fascaplysin derivatives, and binding mechanism analysis, some valuable hints for the discovery of antitumor drug candidates could be provided.

## Introduction

Currently, cancer has gradually become the biggest killer of human health, making the discovery of novel therapies for cancer with hypotoxicity and high efficiency a hot issue in many disciplines (Peto, 2001; Hornak et al., 2006; Willyard, 2016; Deb et al., 2018). Nowadays, numerous studies have shown that various cancers are considered to be related to the cell cycle mainly by the following two important reasons (Jeong et al., 2018): 1) the proliferation of normal cells can be regulated by the indication of growth stimulus and mitotic signals (Malumbres and Barbacid, 2009); 2) the proliferation of tumor cells cannot be normally regulated because of dysregulation of cell cycle (Senderowicz, 2002; Vijayaraghavan et al., 2018; Grant et al., 2019). In addition, protein kinases are reported to be involved in the process of abnormal division during cell proliferation (Leopold et al., 2018). Among the protein kinases, cyclin-dependent kinases (CDKs) belong to serine/threonine kinases family, and the biological functions of CDKs mainly depend on the specific interactions with regulatory activating partners, the cyclins, which participate in the cell cycle progression and transcription (Palmer et al., 2019). More and more experimental results demonstrate that the deregulation of CDKs could induce various medical conditions, especially various cancers (Nakayama and Nakayama, 2006; Malumbres, 2014; Chotiner et al., 2019), thus making CDKs families gradually become potential therapeutic targets.

At present, CDKs inhibitors have been extensively studied, and some small molecule inhibitors have entered clinical trials (Lynce et al., 2018; O’Leary et al., 2016; Oudah et al., 2019; Roskoski, 2019; Whittaker et al., 2017). Among them, some of the first-generation pan-CDK inhibitors (such as dinaciclib, seliciclib, and flavopiridol) are limited in clinical application because of the considerable toxicities and limited efficacy (Erdo et al., 2017; Moharram et al., 2017; Zocchi et al., 2018). Indeed, the broad-spectrum inhibitory effects against CDKs family not only fail to achieve the desired therapeutic effects but may also be the main reason for their side effects, such as the proliferation of various tumor cells during the specific deletion of CDK2 through siRNA or antisense oligonucleotides (Jorda et al., 2018), which further demonstrate the poor druggability of CDKs inhibitors with low selectivity. The lack of clear understanding of the biological function mechanism of CDKs family is the primary obstacle to the discovery of selective CDK inhibitors. Additionally, some CDK subtypes play vital roles in maintaining the physiological functions (Rajput et al., 2016; Parua et al., 2018), and the inhibition of such members will cause serious side effects, such as myelosuppression, anemia, nausea, and diarrhea. Thus, ascribed shortcomings of pan-CDKs inhibitors indicate that the improved selectivity for specific CDK isoforms will contribute to the successful development of CDKs inhibitors as therapeutic antitumor agents.

**FIGURE 1:**
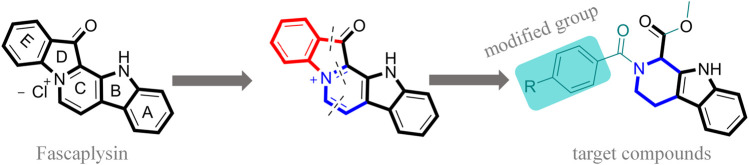
Modification strategy of the target compounds.

Moreover, with the progress of the research, the biological functions of CDK4 have been revealed, and more and more experimental results have proven that the abnormal bioactivities of cyclin D-CDK4/6-INK4-Rb pathway occur in the malignant tumor cells. CDK4 has been reported to be a key protein whose bioactivities are required not only for the emergence of cells from quiescence but also at the G1/S transition in the cell cycle, which involves various cellular misregulations in 60–70% of human cancers (Spring et al., 2016). Thus, selective inhibition of CDK4 with great research prospects is gradually releasing great potential in tumor therapy (Choo and Lee, 2018; Jiang et al., 2019; O’Leary et al., 2016; Pandey et al., 2019). As a marine natural product, fascaplysin can specifically inhibit CDK4 and thus induce G1 arrest and the prevention of pRb phosphorylation of tumor cell lines as well as the normal cell line (Chen et al., 2017; Mahale et al., 2014; Mahgoub et al., 2015; Rath et al., 2018; Yang et al., 2016; Zhidkov et al., 2019). Based on the chemical skeleton (Figure 1), it can be known that fascaplysin has a planar aromatic fused ring structure, and this chemical structure will produce strong cytotoxicity mainly via embedding DNA, changing the conformation of DNA, covalent crosslinking with single or double strands of DNA, or cutting off DNA. Therefore, fascaplysin is often applied as a tool molecule for researching the consequence of CDK4 inhibition, especially in the cell lines with inactivated p16.

In this study, 18 nonplanar tetrahydro-β-carboline analogs (compounds 6a-i and 7a-i) were designed and synthesized by retaining the scaffold of *β*-carboline and breaking the Ring D based on the fascaplysin skeleton (Figure 1), which aimed to reduce the strong cytotoxicity caused by planar structure (Bachurin et al., 2001). In addition, the cytotoxic activities of the synthesized compounds were assessed by MTT assay, and the kinase inhibition test was further adopted to evaluate the inhibitory activities of the title compounds on CDK4. Compound 6c with promising cytotoxic activity against the three cancer cell lines also showed good inhibitory activities against CDK4, and compared to the reference group fascaplysin, the cytotoxicity of compound 6c to the normal cell line was quite weak. In order to better illustrate the inhibitory mechanism of the synthesized nonplanar compounds, computational approaches were comprehensively applied to compare the inhibitory binding modes between the planar fascaplysin and its nonplanar analogs mainly through molecular docking and molecular dynamic (MD) simulation. Finally, the key residues with significant energy contribution to the bindings of fascaplysin and the title compounds were identified and compared by decomposition-free energy contribution, which may provide valuable information for the discovery of novel antitumor agents.

## Materials and Methods

### Chemical Synthesis

#### General Experimental Methods

**FIGURE 2:**
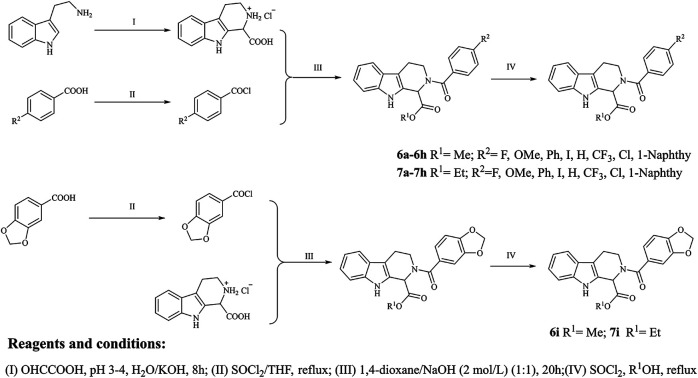
Synthetic route of the target compounds.

The target compounds were synthesized mainly in four steps (Figure 2). All the reaction processes were monitored by thin-layer chromatography (TLC) using silica gel plates (60 F254), and the target compounds were purified by column chromatography, which was performed on silica gel (60–120 mesh) using distilled hexane and ethyl acetate. ^1^H NMR and ^13^C NMR spectra were determined in DMSO-*d*_6_ by using 400 and 100 MHz spectrometers (Instrument Bruke AM-400, 400 MHz), and ESI-Bruker APEXⅡ49e mass spectrometer was applied to record the mass spectra. Perkin-Elmer Spectrum 2000 FTIR was applied to record the infrared spectra. Melting points of the synthesized compounds were measured in open glass capillary tubes with Shengyan electrothermal PIF YRT-3 apparatus without correcting.

#### General Procedure for the Preparation of 2,3,4,9-Tetrahydro-1H-pyrido[3,4-b]indole-1-carboxylic Acid Hydrochloride (Compound 3)

The intermediate compound 3 was synthesized with tryptamine (10 mmol) as the material, and the synthetic routes were as the following steps: 10 mmol tryptophan was dissolved in 100 ml of water and all solids were dissolved by dropping concentrated hydrochloric acid; 40 ml glyoxylic acid was added to the obtained solution, and the pH value of the reaction solution was controlled between 3 and 4 with 10% KOH solution; the mixture was stirred at 0°C for 10 min and stirred at the room temperature for 20 h, which were then acidified with concentrated hydrochloric acid to obtain the solid products; the solid products were washed with water and then dried to obtain the N-substituted tetrahydro-β-carboline acid, which could be converted into 2,3,4,9-tetrahydro-1H-pyrido[3,4-b]indole-1-carboxylic acid hydrochloride in a higher yield (white solid, 90%) due to the good yield of Pictet-Spengler reaction.

#### General Procedure for the Preparation of Substituted Benzoyl Chloride (4a-i)

Substituted benzoic acid (2a-i) was applied as the raw material to synthesize compound 4a-i; the synthetic details were as follows: substituted benzoic acid (2.4 mmol) was dissolved in dry THF, which was treated with dichlorosulfoxide (SOCl_2_, 24 mmol) with pyridine as the catalyst; the reaction mixture was then stirred and refluxed for 1.5 h at 70°C; the solvent and excess SOCl_2_ were removed by reduced pressure distillation, and the obtained products were reserved in a sealed environment.

#### General Procedure for the Synthesis of 2-Benzoyl-2,3,4,9-tetrahydro-1H-pyrido[3,4-b]indole-1-carboxylic Acid (5a-i)

Compound 3 was dissolved into the mixture containing 2 mol/L NaOH solution (2.1 ml) and 1,4-dioxane (5 ml), and benzoyl chloride derivatives were subsequently added to the obtained solution; the mixture was stirred for 10 min at 0°C and then stirred for 20 h at the room temperature; finally, the reaction mixture was acidified with concentrated hydrochloric acid to produce the solid products, which was washed with water and dried to obtain the N-substituted tetrahydro-β-carboline acids (5a-i).

#### General Procedure for the Preparation of Methyl 2-Benzoyl-2,3,4,9-tetrahydro-1H-pyrido[3,4-b]indole-1-carboxylate (6a-i)

N-substituted tetrahydro-β-carboline acids (5a-i) were dissolved into anhydrous methanol, and then 4 ml SOCl_2_ was added to the reaction mixture under −20°C ethanol bath; the reaction solution was stirred vigorously for 1 h under −20°C ethanol bath and then moved to the temperature and stirred for 8 h; the reaction process was monitored by TLC until the reaction was completed, and the solvent was distilled off under reduced pressure; the product was dissolved in ethyl acetate, which was then washed three times with NaHCO_3_ solution and saturated brine, respectively; the title compounds were obtained through filtration and purification using silica gel column chromatography (ethyl acetate/petroleum ether = 1/3).

#### General Procedure for the Preparation of Ethyl 2-Benzoyl-2,3,4,9-tetrahydro-1H-pyrido[3,4-b]indole-1-carboxylate (**7a-i**)

N-substituted tetrahydro-β-carboline acids (**5a-i**) were dissolved into anhydrous ethanol, and then 4 ml SOCl_2_ was added to the reaction mixture under −20°C ethanol bath; the reaction solution was stirred vigorously for 1 h under −20°C ethanol bath, which was then moved to the room temperature and stirred for 8 h; the reaction process was monitored by TLC until the end of the reaction process, and the solvent was distilled off under reduced pressure; the product was dissolved in ethyl acetate, which was then washed three times with NaHCO_3_ solution and saturated brine, respectively; the title compounds were obtained through by filtration and purification using silica gel column chromatography (ethyl acetate/petroleum ether = 1/3).

### Bioassays

#### Cytotoxicity Assay

The cytotoxicity of the target compounds was evaluated via MTT assay; A549, HeLa, HepG2, and WI-38 were selected for the experiment; and the selected cell lines were purchased from Lanzhou Weihuan Biotechnology Development Co., Ltd. All cell lines were maintained in RPMI-1640 medium supplemented with 10% fetal calf serum under the environment of 5% CO_2_ concentration and 37°C. The incubated tumor cells were seeded in the prepared 96-well plates, respectively, to make them attach for 12 h. Afterward, the cells were processed with the synthesized compounds with different concentrations as well as fascaplysin as the reference group for 48 h. After 48 h, 10 μl of MTT solution (5 mg/ml in PBS) was added into each well and incubated for 4 h. Then, the medium was removed and 100 μl DMSO was added to dissolve the blue-colored formazan. The absorbance was detected at 570 nm by microplate reader to calculate the inhibition rate (%) and the IC_50_ was defined using *GraphPad Prism Software* (*version 5.02*) (shown in Table 1).

**TABLE 1:** Cytotoxicity of compounds against cells *in vitro* for 48 h.

Compounds	R^1^	R^3^	Cytotoxicity (IC_50_, μM)*^a^*			
			A549	HeLa	HepG2	WI-38
6a	Me-	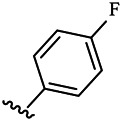	38.28 ± 2.53	28.41 ± 1.12	31.63 ± 2.53	252.74 ± 33.16
6b		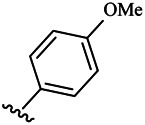	13.25 ± 1.21	14.11 ± 2.69	26.28 ± 1.88	267.39 ± 28.85
6c		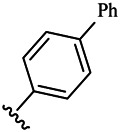	6.53 ± 1.65	1.03 ± 0.19	5.21 ± 0.58	311.51 ± 56.06
6d		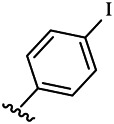	10.84 ± 1.17	19.37 ± 2.56	33.42 ± 5.18	373.27 ± 62.78
6e		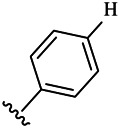	15.53 ± 1.26	16.81 ± 4.12	31.84 ± 2.86	117.68 ± 12.93
6f		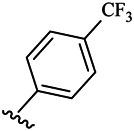	38.71 ± 7.25	29.85 ± 4.73	35.79 ± 3.23	125.05 ± 26.07
6g		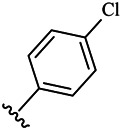	25.74 ± 8.78	22.85 ± 2.31	13.68 ± 2.13	375.43 ± 60.03
6h		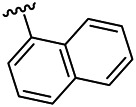	12.66 ± 1.20	10.75 ± 2.69	19.48 ± 0.43	185.66 ± 12.36
6i		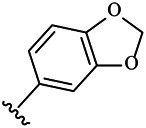	9.60 ± 3.22	3.57 ± 0.93	12.23 ± 3.01	246.54 ± 10.93
7a	Et-	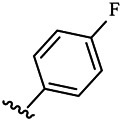	39.53 ± 4.98	29.62 ± 7.07	47.49 ± 4.11	255.43 ± 86.30
7 b		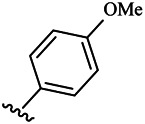	15.48 ± 13.40	15.36 ± 3.10	41.46 ± 2.42	429.00 ± 38.87
7c		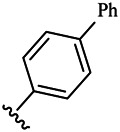	10.45 ± 1.50	4.71 ± 0.70	10.85 ± 1.49	281.09 ± 7.81
7d		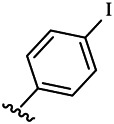	20.35 ± 2.68	27.58 ± 2.38	28.20 ± 0.95	183.46 ± 17.90
7e		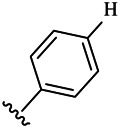	27.28 ± 6.50	19.92 ± 2.45	21.36 ± 1.69	99.82 ± 9.97
7f		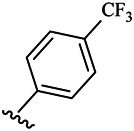	42.12 ± 7.60	47.04 ± 4.80	16.19 ± 3.72	182.69 ± 17.86
7g		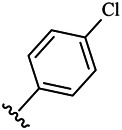	27.28 ± 2.40	24.92 ± 3.44	41.36 ± 1.69	199.82 ± 9.97
7h		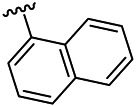	25.48 ± 3.92	16.49 ± 6.64	17.50 ± 13.77	182.65 ± 13.20
7i		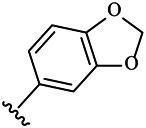	19.96 ± 5.57	6.18 ± 2.78	23.20 ± 9.17	256.15 ± 55.98
Fascaplysin*^b^*			1.05 ± 0.08	0.56 ± 0.05	0.24 ± 0.04	1.86 ± 0.17

^a^ IC_50_ values were calculated from three independent experiments.

^b^ Used as a reference.

#### CDK4 Kinase Inhibition Assay

In this study, the inhibitory activities of the synthesized compounds against CDK4/CycD3 were also evaluated using mobility shift assay, and fascaplysin was used as the positive control compound. CDK4/CycD3 was from Cama, kinase substrate peptide was from GL, and fascaplysin was from Selleckchem. First, the kinase base buffer should be prepared (50.0 mmol/L HEPES, pH 7.5, 5 mM MgCl_2_, 1 mmol DTT, 0.0015% Brij-35). Second, 50 μg CDK4 kinase was added to the kinase base buffer to prepare the kinase solution. The kinase base buffer was used to prepare ATP and substrate peptide solutions, and the obtained solutions were mixed. Third, the synthesized compounds as well as the fascaplysin were diluted with DMSO to the desired concentrations. Fourth, the reaction mixture was incubated at 30°C for 40 min, and 25 μl stop buffer was added to stop the reaction. Finally, conversion was read by Caliper, and GraphPad Prism 5.0 was used to calculate the IC_50_ values of the compounds against enzyme bioactivities (Table 2).

**TABLE 2:** Kinase inhibition profile of the synthesized compounds against CDK4.

Compounds	R^1^	R^3^	IC_50_ *^a^*, μM
			CDK4/CycD3
6a	Me-	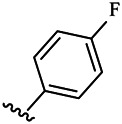	29.56 μM
6b		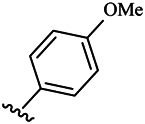	25.68 μM
6c		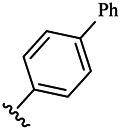	1.89 μM
6d		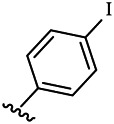	30.28 μM
6e		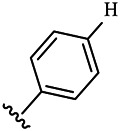	20.68 μM
6f		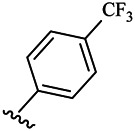	39.58 μM
6g		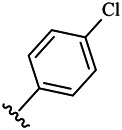	27.46 μM
6h		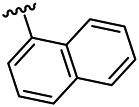	12.38 μM
6i		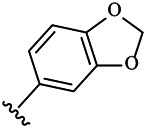	3.38 μM
7a	Et-	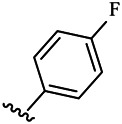	31.56 μM
7b		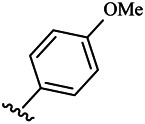	30.68 μM
7c		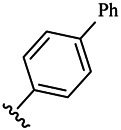	4.89 μM
7d		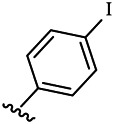	35.28 μM
7e		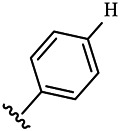	29.68 μM
7f		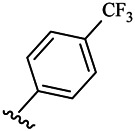	43.89 μM
7g		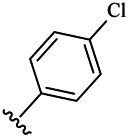	30.42 μM
7h		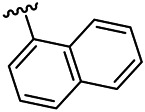	16.38 μM
7i		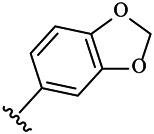	9.38 ± 1.79 μM
Fascaplysin^*b*^			0.79 ± 0.72 μM

^a^ The data represent the mean values of three independent experiments.

^b^ Used as the positive control group.

### Molecular Modeling Studies

#### Molecular Docking

Ligand docking mainly consisted of two procedures: 1) Protein preparation. Currently, the crystal structures of CDK4 have been available from *Protein Data Bank* (PDB), and the crystal structure with ligands can give the researcher valuable instructional suggestion when determining the binding site and defining the docking grid box. The receptor protein first needed to be processed by Protein Preparation Wizard module embedded in *Maestro* after being downloaded from PDB, including assigning bond orders, adding hydrogens, creating zero-order bonds to metals, and creating disulfide bonds. OPLS-2005 was applied during the restrained minimization, and the convergence would reach when the RMSD of the heavy atoms of the protein was of 0.30 Å. The processed protein conformation was used for the subsequent grid generation using the Receptor Grid Generation tool in *Glide* module with the original ligand molecule as reference (Glide v. 5.5, 2005). 2) Ligand preparation. Molecular structures of the title compounds were obtained using *ChemBioDraw Ultra 14.0* and stored in *.sdf format, which were further processed by *LigPre* module using OPLS_2005 as the force field, including ionization and stereoisomers (Epik v. 2.0, 2009). Finally, ligand docking was performed after the preparation of the receptor and ligand. During the docking process, there were 5,000 poses generated after the docking calculation in the initial phase, and the best 400 poses were selected to minimize the energy through conjugate gradient minimizations. The obtained docking poses of the four studied systems, with the most similar orientation with the original ligand in the receptor proteins, were chosen as the initial conformation for molecular modeling.

#### Molecular Dynamic Simulation

MD simulations of the constructed systems were conducted via GPU-accelerated PMEMD in *AMBER16* on 16 cores of an array of two 2.6 GHz Intel Xeon E5-2650v2 processors and 4 pieces of the NVIDIA Tesla K40C graphic card (AMBER v. 16, 2016). First, all the components of the systems (especially the receptor and ligands) were dealt with AmberTools to generate the corresponding coordinate files (*.inpcrd) and topology files (*.prmtop), and the AMBER *ff14SB* (Hornak et al., 2006) and general Amber *force fields* were selected for protein and the ligands, respectively. The Li/Merz ion parameters for SPC/E water model were directly adopted from previous publications. A*ntechamber* was selected to assign the charges of the studied ligands through the restrained electrostatic potential partial charges, and *Gaussian 09* was adopted to optimize the geometry and calculate the electrostatic potential calculations at HF/6–31G* level. Then, the prepared systems were subjected to initial energy minimization through two procedures before molecular dynamic simulation. The solute atoms were processed by harmonic restraint in the first procedure (force constant = 10 kcal mol^−1^⋅Å^−2^), and the atoms were released to move freely in the second procedure. During these two procedures, the energy minimization was conducted by the steepest descent approach for the first 5,000 steps and the subsequent 5,000 steps were processed by the conjugate gradient method. Then, the four prepared systems were heated from 0 K gradually to 100 K and progressively to 310 K with the protein restrained over 100 ps in the NVT ensemble. Subsequently, the equilibration of the prepared systems’ periodic boundary conditions was reached via conducting 5 ns unrestrained equilibration at 310 K. Finally, the unrestrained 500 ns MD simulations were performed for the four prepared systems in the NPT ensemble under the temperature of 310 K and the pressure of 1 atm. The temperature was controlled by Langevin dynamics and pressure was controlled using *Monte Carlo barostat* (Zheng et al., 2017; Xue et al., 2018b; Yang et al., 2018).

In all simulations, *Particle-mesh Ewald* (PME) was adopted to process the long-range electrostatic interactions, and the *SHAKE algorithm* (Larini et al., 2007) was used to maintain all bonds rigid. During the MD simulation, 2.0fs time step and 10.0 Å cutoff for the nonbonded interactions were selected as the parameters. All the analyses of MD trajectories, including RMSD, the representative structures, and binding free energy, were conducted by *cpptraj* and *mm_pbsa.pl* programs available in *AMBER16*, and *PyMOL* software was used to visualize the conformations of the studied systems (Schrodinger, 2019).

#### Thermodynamic Calculations

In this study, the binding free energy and per-residue decomposed energy were calculated by the following equations:

$${\text{ΔG}}_{\text{MM}/\text{GBSA}}={\text{ΔE}}_{\text{vdW}}+{\text{ΔE}}_{\text{ele}}+{\text{ΔG}}_{\text{pol}}+{\text{ΔG}}_{\text{nonpol}},$$ (1)

$${\text{ΔG}}_{\text{calc}}^{\text{per}-\text{residue}}={\text{ΔE}}_{\text{vdW}}^{\text{per}-\text{residue}}+{\text{ΔE}}_{\text{ele}}^{\text{per}-\text{residue}}+{\text{ΔG}}_{\text{pol}}^{\text{per}-\text{residue}}+{\text{ΔG}}_{\text{nonpol}}^{\text{per}-\text{residue}}.$$ (2)

ΔE_*vdW*_ and ΔE_*ele*_ represented the van der Waals interaction and electrostatic contribution in the gas phase, respectively. ΔG_*pol*_ and ΔG_*nonpol*_ are the polar and nonpolar solvent interaction energies. ΔG_*nonpol*_ = 0.0072×ΔSASA was used to calculate ΔG_*nonpol*_ by the linear combination of the pairwise overlap (LCPO) method (Weiser et al., 1999), where SASA represents the solvent accessible area. The definition of ${\text{ΔE}}_{vdW}^{per-residue}$, ${\text{ΔE}}_{ele}^{per-residue}$, and ${\text{ΔG}}_{pol}^{per-residue}$ was similar to that in Eq. 1, and ${\text{ΔG}}_{nonpol}^{per-residue}$ was calculated by recursively approximating a sphere around a given atom based on the icosahedron (ICOSA) method (AMBER v. 16, 2016).

#### Identifying the Hot- and Warm-Spot Residues via Hierarchical Clustering

The clustering tree of residues contributing to the ligands (>0.1 kcal/mol) binding to CDK4 was constructed using *R* statistical analysis package (Tippmann, 2015) with the similarity levels among vectors measured by the *Manhattan* distance:

$$\text{Distance }\left(\text{a},\text{ b}\right)=\sum \left|a_{i}-b_{i}\right|,$$ (3)

where *i* represented the dimensions of the per-residue energies *a* and *b*, and Ward’s minimum variance method was used as the clustering algorithm here, which was applied to minimize the total variance within cluster (Xue et al., 2018a). The online tree generator *iTOL* was used to generate the hierarchical tree graph (Letunic and Bork, 2016). The residues with binding free energy contribution favoring the binding of the ligands were shown in red (the residues with the highest contribution were displayed by exact red and the residues with lower contributions gradually faded toward the white (no contribution)). In addition, the residues were displayed in blue hampered CDK4 binding, and the lower ones gradually faded toward white color.

## Results and Discussion

### Chemistry

The 18 novel tetrahydro-β-carboline analogs were designed and synthesized on the basis of the molecular skeleton of fascaplysin via cyclization and acylation according to the classical Pictet-Spengler reaction (Figure 2). The nonplanar analogs were obtained via breaking the Ring D and retaining Rings A–C of fascaplysin, aiming to reduce the strong toxic and side effects caused by the planar structure. In addition, all the synthesized compounds were characterized by ^1^H NMR, ^13^C NMR, IR, and ESI-MS methods (Supplementary Material).

### Biological Activities

To study the growth-inhibitory effects of the target compounds on cancer cell lines, A549, HeLa, and HepG2 were applied to examine the inhibitory activities of the title compounds in this study using MTT assay, and fascaplysin was also used as the reference group. The cytotoxic activities as 50% inhibitory concentration (IC_50_) values are shown in Table 1, and according to the results of the MTT assay, the synthesized compounds exhibited certain inhibitory activities against the tumor cell lines. Generally, the biological activities of the target compounds with methyl as R^1^ group were superior to those of the derivatives with ethyl substitution, which were negatively correlated with the electronic ability of the *R* ^2^ group based on the structure-activity relationship analysis, especially F-, Cl-, Br-, I-, and CF_3_-substituted compounds. Moreover, it should be noted that compound 6c with *R* ^2^ group as phenyl substitution exerted the best inhibitory activity against HeLa (IC_50_ = 1.03 ± 0.19 μM) (comparable to positive control (IC_50_ = 0.56 ± 0.05 μM), which was related to the introduction of benzene ring increasing the hydrophobic interactions with the target. In addition, all the synthesized compounds showed relatively weak cytotoxicity to normal cell lines (IC_50_ ranged from 99.82 ± 9.97 μM to 429.00 ± 38.87 μM), indicating that such nonplanar compounds were less selective for the normal cells and increased their safety. In addition, the results of kinase inhibition experiments were basically consistent with that of the MTT assay, and the inhibitory activities of compound 6c against CDK4 were the most potent among the synthetic compounds (Table 2), which might be a potential CDK4 inhibitor.

### In Silico Molecular Modeling Studies

#### Construction of the Studied Complexes

**FIGURE 3:**
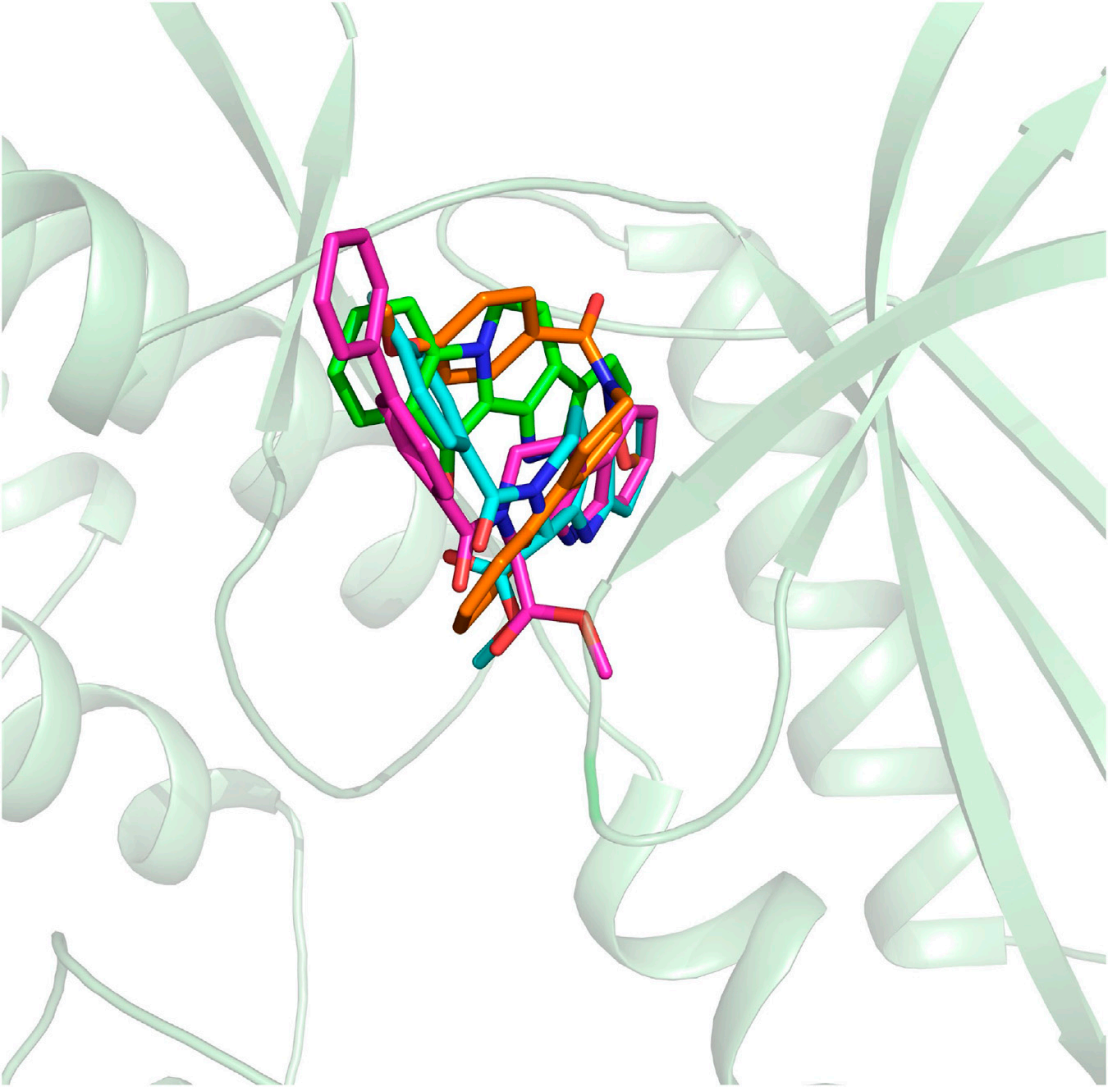
Structural alignment of the initial docking poses of the studied molecules.

On the basis of the resolved protein structure of CDK4 in Protein Data Bank (PDB entry: 2W96) (Day et al., 2009), the molecular docking approach was adopted to determine the initial conformations of the synthesized compounds and fascaplysin in CDK4, and the docking scores and the spatial similarity levels to the CDK4 inhibitors in the CDK2 (Ikuta et al., 2001) (adjacent protein family of CDK4) were applied as the main criteria for judging the initial conformations. Docking scores provided by *Glide* could be used to evaluate the binding interactions between the ligands and receptors, and the larger absolute value of the docking score indicated the more stable binding of the ligand to the receptor. In this study, the docking scores of the selected initial conformations should be consistent with the inhibitory activities of the corresponding compounds against CDK4; that is, the gradient of the docking scores was consistent with that of the IC_50_ of the compounds. As shown in Figure 3, the active pocket of CDK4 mainly consists of *β*-sheet chains, and the binding site was relatively narrow. From the superimposition of the obtained initial conformations, the docked ligands were consistent overall in space with Rings A, B, and C penetrating into the active pocket, and the detailed interactions between the docked ligands and CDK4 also needed further research and identification by MD simulation.

#### Assessing the Stability of the Molecular Dynamic Simulation via RMSD Analysis

**FIGURE 4:**
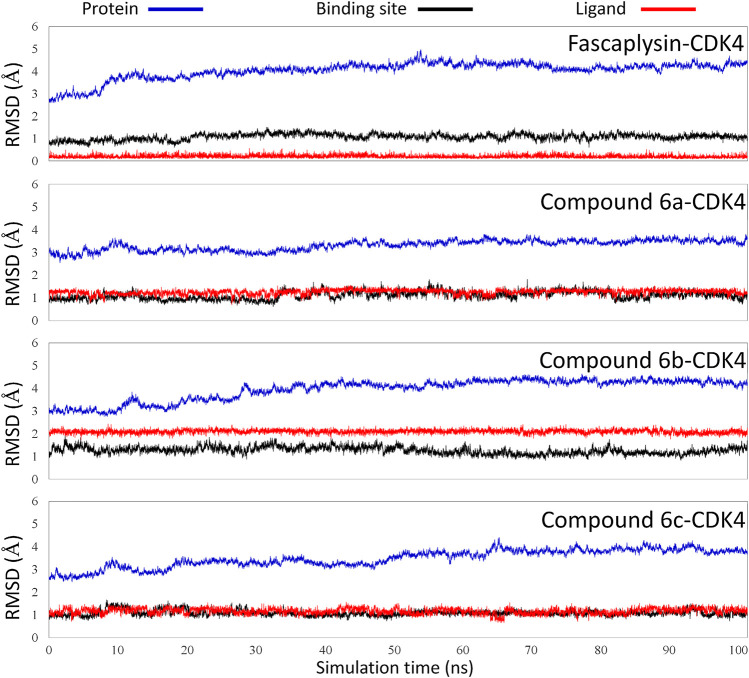
Root mean square deviations of protein backbone atoms, ligand heavy atoms, and binding site residue backbone atoms as a function of time in MD simulations.

**FIGURE 5:**
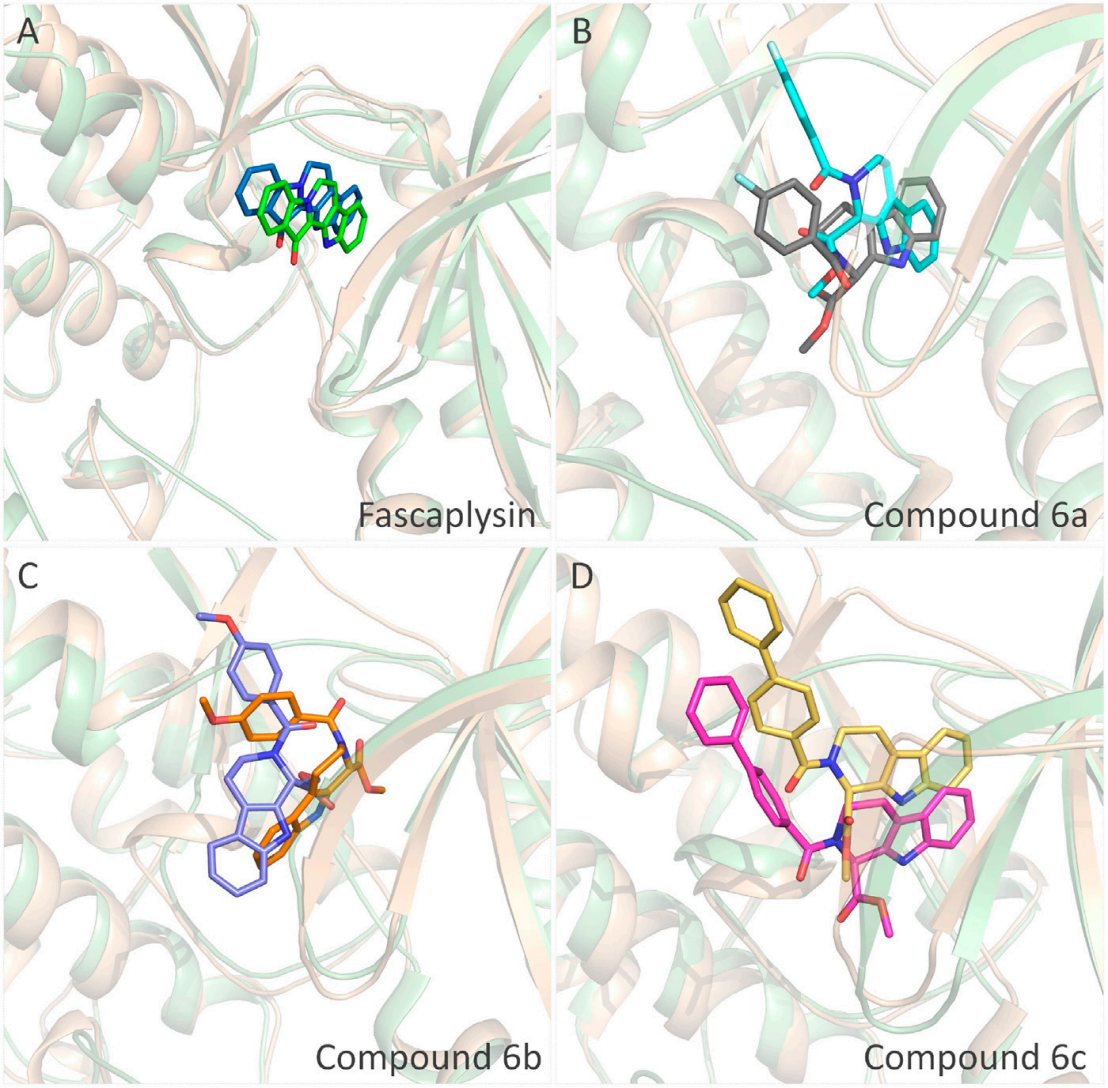
Structural superimposition of the initial docking poses and the representative conformations of all the studied systems: **(A)** initial conformation (green) and representative snapshot (sky blue) of fascaplysin in CDK4; **(B)** initial conformation (cyan) and representative snapshot (gray) of compound 6a in CDK4; **(C)** initial conformation (orange) and representative snapshot (slate) of compound 6b in CDK4; **(D)** initial conformation (magentas) and representative snapshot (yellow) of compound 6c in CDK4.

The obtained initial complexes were further assessed by 100 ns MD simulation, and the RMSD values of the skeleton atoms of CDK4, the residues consisting of the active pocket, and the docked ligands were chosen to monitor the equilibrium of the dynamic process. Based on the RMSD values (Figure 4), all the studied systems could reach the equilibrium state with slight fluctuation at 50 ns, and an additional 50 ns MD simulations were performed to guarantee the continuous and stable equilibration of the four studied systems. According to the RMSD values of ligand (colored by red), fascaplysin and compound 6c had less volatility during the MD simulation process, which was consistent with superimposition of the initial conformation and representative conformation of the studied systems (Figures 5A,D). In addition, the molecular dynamics equilibrium trajectories between 50 ns and 100 ns were adopted for subsequent energy analysis, especially binding free energy and decomposition-free energy.

#### Identification of the Hot-Spot Residues for Target-Ligand Interaction

**FIGURE 6:**
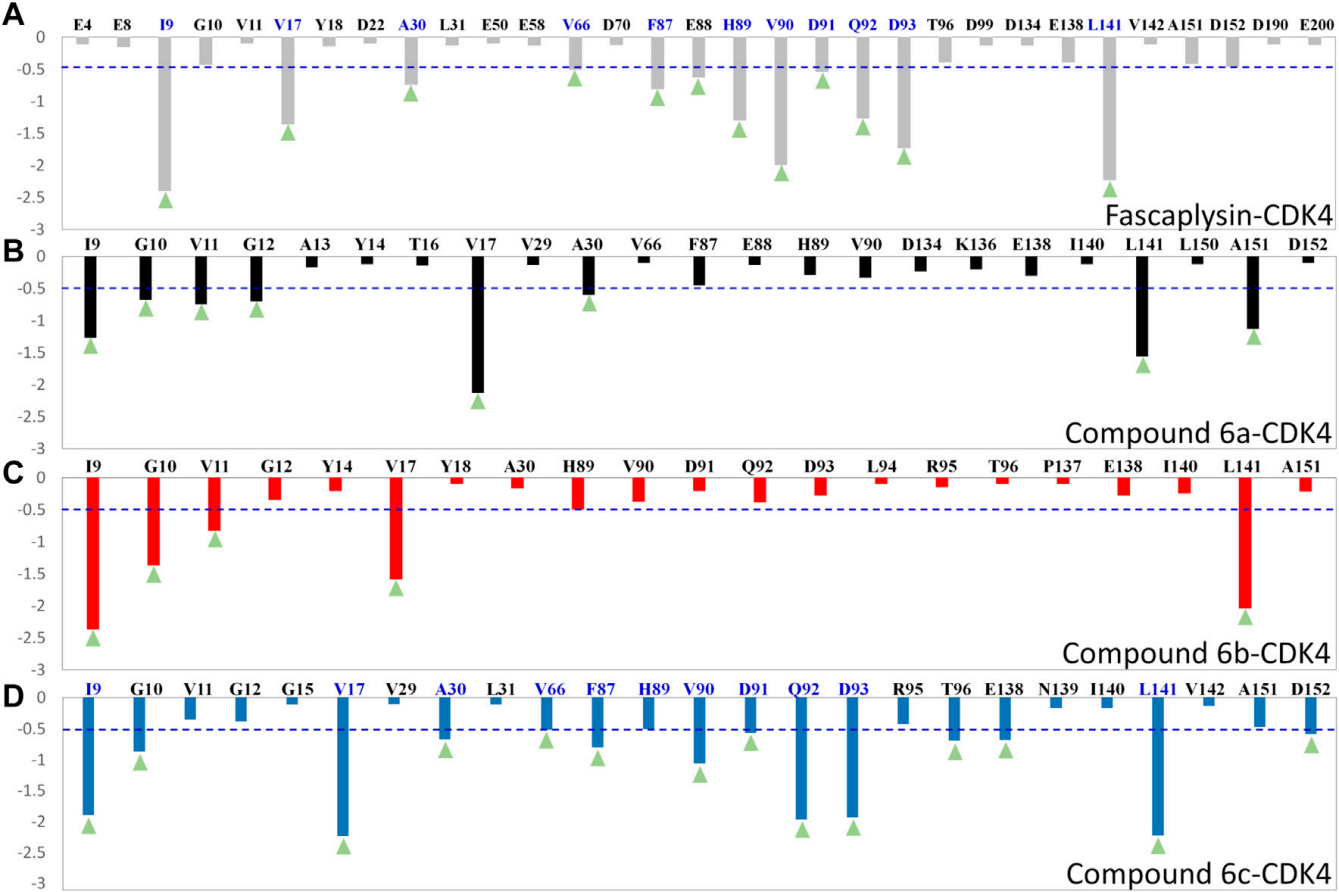
Per-residue binding free energy decomposition of the residues with energy contribution: **(A)** fascaplysin-CDK4 system; **(B)** compound 6a-CDK4 system; **(C)** compound 6b-CDK4 system; **(D)** compound 6c-CDK4 system.

To quantify the key amino acids contributing to the interactions between the docked ligands and receptor, the residues with the decomposition-free energy (≥0.1 kcal/mol) to the compounds’ binding were identified. According to Figure 6, the energy contribution of the same amino acid in CDK4 to the different docked ligands varied greatly (taking VAL90 in CDK4 as an example, it contributed −1.99, −1.03, −0.38, and −0.33 kcal/mol to the binding of fascaplysin, compound 6c, compound 6b, and compound 6a, respectively), and different amino acids had different decomposition-free energy contribution (taking compound fascaplysin in CDK4 as an example, the energy contribution of LEU141 equaled −2.23 kcal/mol, nearly 20 times of GLU4’s contribution (−0.11 kcal/mol)) (Figure 6A). Among the selected residues, the amino acids with significant energy contribution (≥0.5 kcal/mol) were identified as the hot-spot residues, which were labeled by light green triangle. Based on Figure 6, it could be learned that the number of labeled amino acids varied largely among all the studied systems, with 12, 8, 5, and 14 labeled amino acids in the fascaplysin-CDK4, compound 6a-CDK4, compound 6 b-CDK4, and compound 6c-CDK4, respectively. In addition, the amino acids that contributed significantly to the compound 6c′s binding were highly consistent with those that contributed significantly to fascaplysin’s binding, including I9, V17, A30, V66, F87, H89, V90, D91, Q92, D93, and L141 (Figures 6A,D).

**FIGURE 7:**
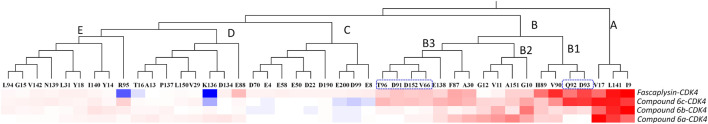
Key residues were identified by hierarchically clustering per‐residue energy contributions across four systems, and the residues were mainly divided into five Chains, namely Chain **A–E**. Residues favoring the binding of ligands were colored in red; residues hampering the binding of ligands were shown in blue (the highest one was colored as standard blue and the lower one was set to fade gradually to white); the white color here represented the residues with no energy contribution.

Moreover, per-residue energy contributions of the residues in CDK4 to the binding of the synthesized compounds and fascaplysin were hierarchically clustered, and the key residues were further compared (Zhang et al., 2019). The residues with energy contribution were divided into five chains, namely, Chains A–E (Figure 7), and the residues located in Chain A were identified as the hot-spot residues, which largely facilitated the bindings of the studied ligands. As shown in Figure 7, Chain B was further divided into Chains B1–3; the amino acids clustered into Chain B_1_ had a large difference in the energy contribution for the studied compounds’ binding. In addition, the energy contributions of the amino acids classified into Chain A and Chain B were basically consistent for fascaplysin and compound 6c’s binding, but the energy contributions of amino acids located in Chain B for compound 6a and 6b’s binding were fewer and resulted in the significant decrease in their binding free energies, which might be the main reason for their relatively low inhibitory activities against CDK4.

#### Comparison of the Inhibitory Binding Modes Between the Planar Fascaplysin and Its Nonplanar Tetrahydro-β-carboline Analogs in CDK4

**FIGURE 8:**
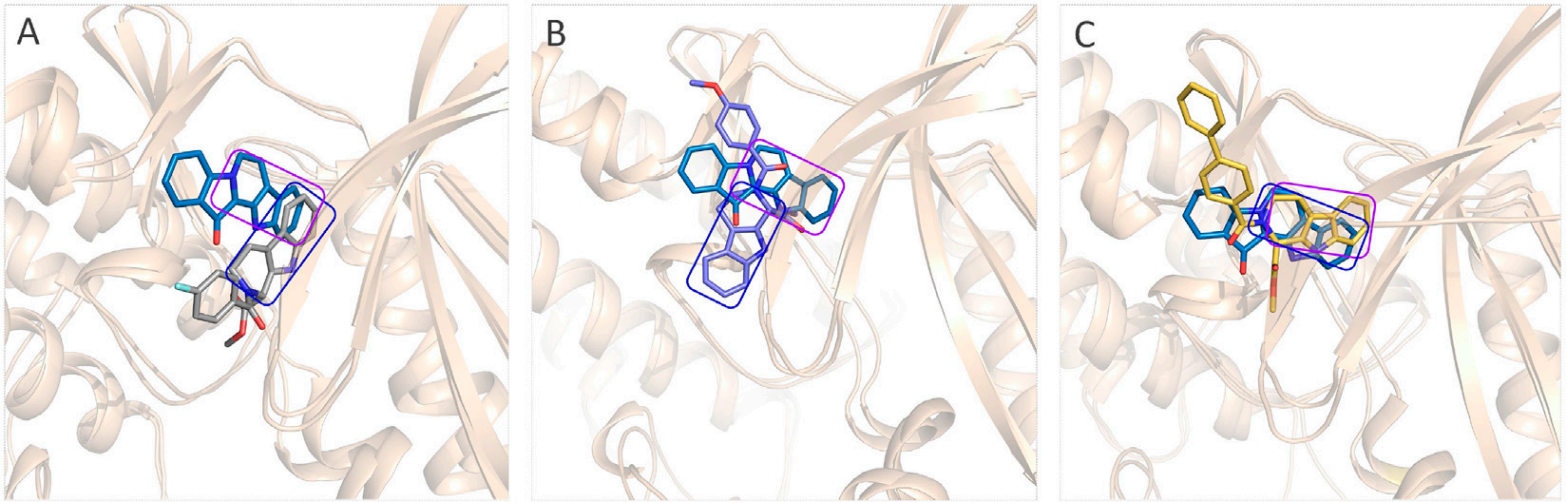
Comparison between the binding modes of the synthesized compounds and that of fascaplysin: **(A)** alignment of CDK4-compound 6a and CDK4-fascaplysin; **(B)** alignment of CDK4-compound 6b and CDK4-fascaplysin; **(C)** alignment of CDK4-compound 6c and CDK4-fascaplysin.

The representative conformations of the four studied systems were extracted from the equilibrium trajectories between 50 ns and 100 ns, which were aligned to their corresponding initial conformations (Figure 5). According to Figures 5A,D, it could be found that fascaplysin and compound 6c had relatively little conformational changes in the binding pockets during the MD process, which could bind stably in their binding sites. However, compound 6a and compound 6 b had relatively large fluctuations in their binding sites (Figures 5B,C), which was related to their interactions with the amino acids in the binding sites. What is more, the systems complexed with compounds 6a–6c were superimposed into the fascaplysin system to compare their binding conformations with that of fascaplysin, respectively. Through the structural superimposition, compound 6a and compound 6b had relatively large spatial differences with fascaplysin at the docking site, especially the functional group, three-ring structure (Figures 8A,B). According to Figure 8C, the spatial orientation of compound 6c was highly consistent with that of fascaplysin at the binding pocket, which might be the main reason for its higher inhibitory activities than that of the other two synthesized compounds.

**FIGURE 9:**
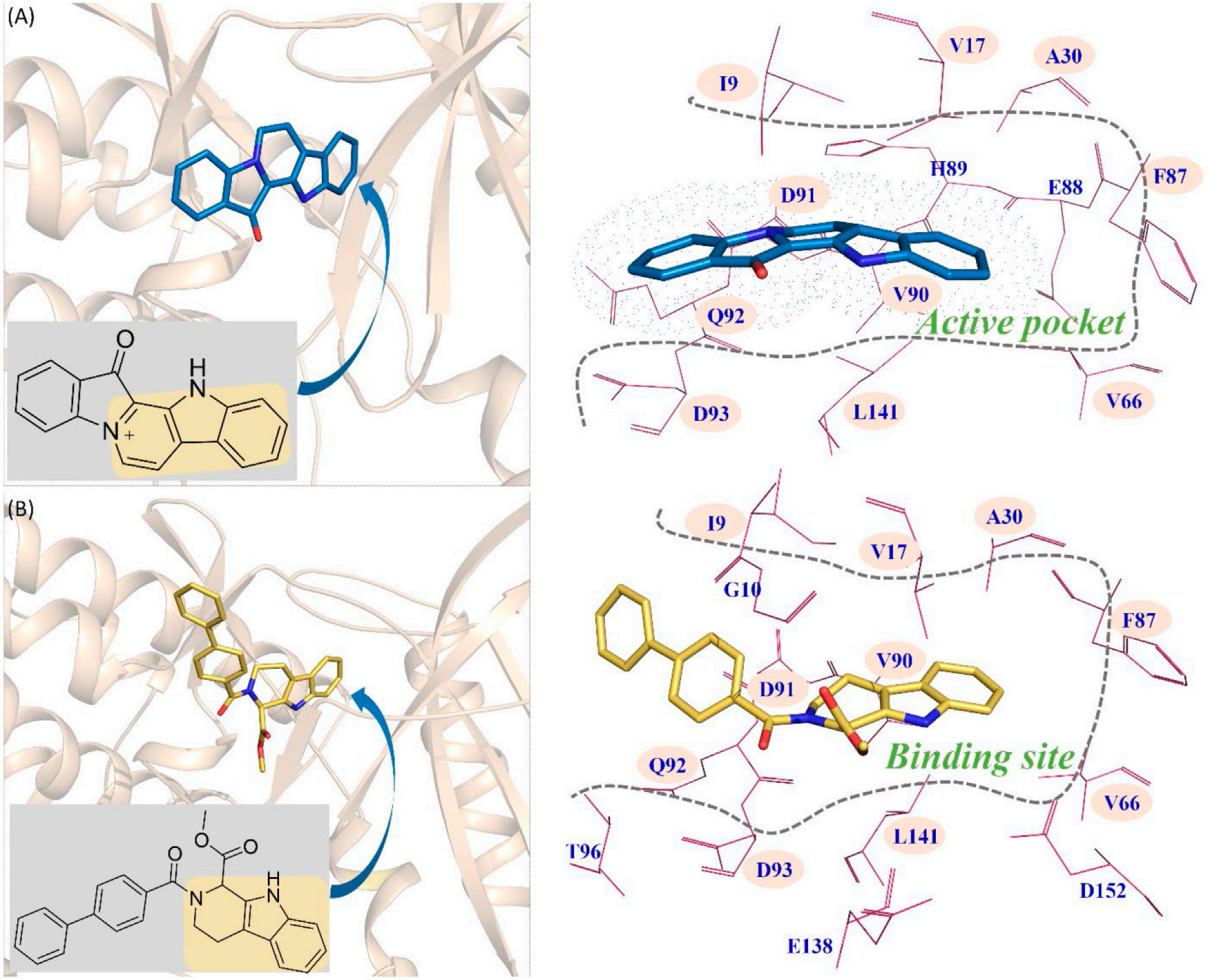
Comparison between the key residues consisting of the binding site of compound 6c and that of fascaplysin **(A)** fascaplysin in CDK4; **(B)** compound 6c in CDK4.

From Figure 9A, the functional group of fascaplysin went deep into the active pocket, and the active pocket was mainly composed of the following amino acids with significant energy contribution (≥0.5 kcal/mol): I9, V17, A30, V66, F87, E88, H89, V90, D91, Q92, D93, and L141. In addition, the binding site of compound 6c consisted of 14 residues, namely, I9, G10, V17, A30, V66, F87, V90, D91, Q92, D93, T96, E138, L141, and D152 (Figure 9B). Via comparing the docking site of compound 6c and fascaplysin, 10 residues that formed their binding site were consistent, namely, I9, V17, A30, V66, F87, V90, D91, Q92, D93, and L141, highlighted by the pink background, which further confirmed that the binding site of compound 6c was highly consistent with that of fascaplysin. However, as for the other two systems (CDK4-compound 6a and CDK4-compound 6b), there were only three and four identified key residues that strongly interacted with compounds 6a and 6b.

#### Comparison of the Synthesized Compounds

The synthesized compounds aimed to break the planarity of fascaplysin via modifying the Ring D and retained the remaining three rings, which intended to reduce DNA toxicity. Compounds 6a, 6b, and 6c and fascaplysin with bioactivities gradient were selected as molecular probes to explore the difference between the binding modes of nonplanar tetrahydro-β-carboline analogs and planar fascaplysin. Through molecular dynamics simulation, it was found that the Rings A, B, and C of fascaplysin could penetrate deep into the active pocket and stably bind at the active site, thereby exerting strong inhibitory activity. Due to the difference in *R* ^2^ groups of the synthesized compounds, the functional groups (A, B, and C rings) differed greatly in the active pocket. Among them, the three-ring structures of compound 6a and compound 6b were biased out of the active pocket. Compound 6c, due to the larger biphenyl substituent, was able to form strong interactions with amino acids D91, Q92, and D93, which played a key role in maintaining the conformation in the active pocket and ultimately ensured its strong inhibitory activity against CDK4.

## Conclusion

In conclusion, 18 nonplanar tetrahydro-β-carboline analogs were designed and synthesized on the basis of the molecular skeleton of fascaplysin and characterized by ^1^H NMR, ^13^C NMR, IR, and ESI-MS diffraction methods. The biological activities of all the synthesized compounds were evaluated at the cellular and kinase level. From the MTT assay, the designed compounds exhibited varying degrees of proliferation inhibition against the selected tumor cell lines, especially the HeLa cell line. Generally, the biological activities of the target compounds with methyl as R^1^ group were superior to those of the derivatives with ethyl substitution, which were negatively correlated with the electronic ability of the *R* ^2^ group based on the structure-activity relationship analysis. Compound 6c with *R* ^2^ group as phenyl substitution exerted the best inhibitory activity, which was related to the introduction of benzene ring increasing the hydrophobic interactions with the target. It should be noted that the synthesized compounds showed quite weak toxicity to the normal cell line, indicating that the destruction of the planar structure did reduce the cytotoxicity to normal cells. Based on the molecular modeling studies, compound 6c with bulky biphenyl substituent could bind stably in the active pocket and it could be found that Rings A–C of compound 6c were highly consistent with those of fascaplysin in the spatial conformation. Such modification strategy could not only break the planarity of the molecular framework and reduce the adverse effects but also ensure the stable binding to CDK4, providing valuable insights for the design of novel CDK4 inhibitors.

## Data Availability

The datasets presented in this study can be found in online repositories. The names of the repository/repositories and accession number(s) can be found in the article/Supplementary Material.
