# Supplementary material for: Comparison of the Inhibitory Binding Modes Between the Planar Fascaplysin and Its Nonplanar Tetrahydro-β-carboline Analogs in CDK4
Source: Front Chem. 2021 Feb 18;9:614154. doi: 10.3389/fchem.2021.614154 (PMC7930575; doi:10.3389/fchem.2021.614154)
Supplement: Supplementary file 1 [file table1.docx]

**Comparison of the Inhibitory Binding Modes between the Planar Fascaplysin and its Non-planar Tetrahydro-β-carboline Analogs in CDK4**

**Yan Liang ^1^** ^†^**, Huili Quan** **^2^** ^†^**, Tong Bu ^3^, Xuedong Li ^3^, Xingang Liu ^3^, Songsong Wang ^4^, Dian He ^1 *^, Qingzhong Jia ^3 *^, Yang Zhang ^3 *^**

**^1^** Materia Medica Development Group, Institute of Medicinal Chemistry, Lanzhou University School of Pharmacy, Lanzhou 730000, China

**^2^** The Fourth Hospital of Shijiazhuang, Shijiazhuang 050017, China

**^3^** Department of Pharmacology, Hebei Medical University, Shijiazhuang 050017, China

**^4^** The Second Hospital of Hebei Medical University, Shijiazhuang 050017, China

**^†^** *These authors contributed equally to this work*.

**^*^** Corresponding author: Prof. Dian He, Materia Medica Development Group, Institute of Medicinal Chemistry, Lanzhou University School of Pharmacy, Lanzhou 730000, China. E-mail: [hed@lzu.edu.cn](mailto:hed@lzu.edu.cn);

Prof. Qingzhong Jia, Department of Pharmacology, Hebei Medical University, Shijiazhuang 050017, China. E-mail: qizhjia@ hebmu.edu.cn.

Dr. Yang Zhang, Department of Pharmacology, Hebei Medical University, Shijiazhuang 050017, China. E-mail: zhangyang_16@hebmu.edu.cn.

**Supplementary information for the spectra data of the synthesized compounds**

*Spectra data*

Methyl-2-(4-fluorobenzoyl)-2, 3, 4, 9-tetrahydro-1*H*-pyrido [3, 4-b] indole-1-carboxylate (Compound 6a)

White solid, yield 65%, m.p.: 182.8~183.7 ℃. IR (KBr) v (cm^-1^): 3379 (N-H), 1756 (C-C=O), 1634 (N-C=O), 1235 (C-F); ^1^H NMR (400 MHz, DMSO-*d_6_*): δ 11.07 (s, 1H), 7.51 – 7.75 (m, 2H), 7.32 – 7.48 (m, 4H), 7.12 (t, *J* = 7.4 Hz, 1H), 7.01 (t, *J* = 7.4 Hz, 1H), 6.04 (s, 1H), 3.94 (d, *J* = 9.5Hz, 1H), 3.79 (s, 3H), 3.48 – 3.66 (m, 1H), 2.67 – 2.99 (m, 2H); ^13^C-NMR (100MHz, DMSO-*d_6_*, TMS, ppm): δ 170.4, 169.4, 162.2, 137.0, 132.2, 130.1, 127.0, 126.2, 122.2, 119.3, 118.5, 116.3, 112.1, 109.0, 65.5, 53.3, 46.6, 20.9; ESI-MS: Calcd. for C_20_H_17_FN_2_O_3_ [M + Na]^+^ 375.3654; Found: 375.1130.

Methyl-2-(4-methoxybenzoyl)-2, 3, 4, 9-tetrahydro-1*H*-pyrido [3, 4-b] indole-1-carboxylate (Compound 6b)

White solid, yield 78%, m.p.: 200.8~201.6℃. IR (KBr) v (cm^-1^): 3213 (N-H), 1754 (C-C=O), 1621 (N-C=O), 1274 (Ar-O-CH3); ^1^H-NMR (400 MHz, DMSO-*d_6_*): δ 11.06 (s, 1H), 7.45 (td, *J* = 17.4, 16.4, 7.7Hz, 4H), 7.06 (ddt, *J* = 22.4, 14.9, 7.3Hz, 4H), 5.98(s, 1H), 4.04 (d, *J* = 12.0Hz, 1H), 3.81(d, *J* = 19.1Hz, 6H), 3.46–3.66 (m, 1H), 2.67-2.99 (m, 2H). ^13^C-NMR (100MHz, DMSO-*d*_6_, TMS, ppm): δ 171.2, 169.7, 161.2, 137.0, 129.6, 127.6, 127.2, 126.2, 122.2, 119.3, 118.5, 114.4, 112.1, 109.0, 65.5, 55.8, 53.4, 47, 21.6. ESI-MS: Calcd. for C_21_H_20_N_2_O_4_ [M + Na]^+^ 387.1423; Found: 387.1345.

Methyl-2-([1, 1’-biphenyl]-4-carbonyl)-2, 3, 4, 9-tetrahydro-1*H*-pyrido [3, 4-b] indole-1 carboxylate (Compound 6c)

White solid, yield 80%, m.p.: 177.8~179.6℃. IR (KBr) v (cm^-1^): 3357 (N-H), 1778 (C-C=O), 1654 (N-C=O). ^1^H NMR (400 MHz, DMSO-*d_6_*): δ 11.09 (s, 1H), 7.82 (d, *J* = 7.9 Hz, 2H), 7.74 (d, *J* = 7.7 Hz, 2H), 7.61 (d, *J* = 7.8 Hz, 2H), 7.54–7.39 (m, 5H), 7.07 (dt, *J* = 44.2, 7.4 Hz, 2H), 6.09 (s, 1H), 4.03 (dq, *J* = 9.3, 3.8, 3.2 Hz, 1H), 3.74 –3.41 (m, 1H), 3.35 (s, 1H), 2.98 – 1.98 (m, 3H), 1.92 – 0.51 (m, 2H). ^13^C-NMR(100MHz, DMSO-*d_6_*, TMS, ppm): δ 171.08, 169.52, 142.32, 139.70, 137.04, 134.08, 129.54, 128.48, 128.16, 127.39, 127.31, 127.05, 126.24, 122.19, 119.32, 118.52, 112.13, 108.93, 53.20, 44.60, 26.81, 21.58. ESI-MS: Calcd for C_26_H_22_N_2_O_3_ [M + H]^+^ 411.1630; Found: 411.1712.

Methyl-2-(4-iodobenzoyl)-2, 3, 4, 9-tetrahydro-*1H*-pyrido [3,4-b] indole-1-carboxylate (Compound 6d)

White solid, yield 73.2%, m.p.:195.60~196.50℃. ^1^H NMR (400 MHz, DMSO-d6) δ 11.06 (s, 1H), 7.90 (d, *J* = 7.9 Hz, 2H), 7.43 (dd, *J* = 13.4, 8.0 Hz, 2H), 7.31 (d, *J* = 8.0 Hz, 2H), 7.12 (t, *J* = 7.6 Hz, 1H), 7.01 (t, *J* = 7.4 Hz, 1H), 6.05 (s, 1H), 4.13 – 3.87 (m, 1H), 3.79 (s, 3H), 3.61 – 3.41 (m, 1H), 2.76 (d, *J* = 3.7 Hz, 2H). ^13^C NMR (100 MHz, DMSO-d6) δ 170.54, 169.39, 138.01, 137.01, 135.20, 129.42, 126.89, 126.19, 122.19, 119.33, 118.51, 112.12, 108.87, 97.64, 53.22, 53.18, 44.53, 21.49. MS(ESI+) m/z :461.2319 [M+H]^+^.

Methyl-2-benzoyl-2, 3, 4, 9-tetrahydro-*1H*-pyrido [3, 4-b] indole-1-carboxylate (Compound 6e)

White solid, yield 53.6%, m.p.: 167-171℃. ^1^H NMR (400 MHz, Chloroform-*d*) δ 8.52 (s, 1H), 7.57 – 7.43 (m, 6H), 7.38 (d, *J* = 8.0 Hz, 1H), 7.12 (t, *J* = 7.5 Hz, 1H), 6.25 (s, 1H), 4.11 (dd, *J* = 13.7, 5.1 Hz, 1H), 3.83 (s, 3H), 3.61 (td, *J* = 13.2, 12.7, 4.0 Hz, 1H), 2.96 – 2.72 (m, 2H).^13^C NMR (101 MHz, Chloroform-*d*) δ 171.94, 169.06, 136.38, 130.19, 128.66, 127.07, 126.41, 126.27, 122.68, 119.86, 118.38, 111.28, 109.79, 52.99, 52.86, 44.47, 21.80. MS(ESI+) m/z: 294.1618 [M+H]^+^.

Methyl-2-(4-(trifluoromethyl)benzoyl)-2, 3, 4, 9-tetrahydro-*1H*-pyrido [3, 4-b] indole-1-carboxylate (Compound 6f)

Light yellow solid, yield 56.3%, m.p:193.3~194.36℃. ^1^H NMR (400 MHz, DMSO-*d_6_*) δ 10.78 (d, *J* = 141.9 Hz, 1H), 7.86 (d, *J* = 7.9 Hz, 2H), 7.69 (t, *J* = 7.3 Hz, 2H), 7.45 – 7.19 (m, 2H), 7.02 (dt, *J* = 33.7, 7.4 Hz, 2H), 4.71 (d, *J* = 125.6 Hz, 2H), 4.46 – 3.93 (m, 1H), 3.53 – 3.26 (m, 1H), 2.97 – 2.62 (m, 2H). ^13^C NMR (100MHz, DMSO-*d_6_*) δ 169.17, 140.80, 136.50, 130.89, 130.10, 128.05, 126.96, 126.05, 125.76, 121.37, 119.07, 118.00, 111.56, 106.99, 45.84, 21.99. MS(ESI+) m/z :403.1265[M+H]^+^.

Methyl 2-(4-chlorobenzoyl)-2,3,4,9-tetrahydro-*1H*-pyrido[3,4-b] indole-1-carboxylate (Compound 6g)

White solid, yield 74.5%, m.p: 200.3~201.5℃. ^1^H NMR (400 MHz, Chloroform-*d*) δ 8.50 (s, 1H), 7.53 – 7.41 (m, 5H), 7.38 (d, *J* = 8.1 Hz, 1H), 7.21 (s, 1H), 7.13 (d, *J* = 7.6 Hz, 1H), 6.21 (s, 1H), 4.07 (dd, *J* = 13.7, 5.1 Hz, 1H), 3.83 (s, 3H), 3.62 (ddd, *J* = 13.6, 11.7, 4.1Hz, 1H),2.94–2.72(m,2H).^13^C-NMR (100 MHz, Chloroform-*d*) 170.89, 168.89, 136.38, 133.84, 129.18, 128.96, 128.62, 128.03, 126.21, 126.19, 122.76, 119.93, 118.40, 111.29, 109.70, 52.94, 44.53, 21.78.

Methyl-2-(2-naphthoyl)-2, 3, 4, 9-tetrahydro-*1H*-pyrido [3, 4-b] indole-1-carboxylate (Compound 6h)

Off-white powder, yield 71.2%, m.p:183.6~184.5℃. ^1^H NMR (400 MHz, Chloroform-*d*) δ 8.57 (s, 1H), 8.04 (s, 1H), 7.94 – 7.85 (m, 3H), 7.66 – 7.43 (m, 5H), 7.38 (d, *J* = 8.1 Hz, 1H), 7.12 (t, *J* = 7.4 Hz, 1H), 6.31 (d, *J* = 1.8 Hz, 1H), 4.17 (dd, *J* = 13.7, 5.1 Hz, 1H), 3.84 (s, 3H), 3.69 – 3.59 (m, 1H), 2.94 (dddd, *J* = 17.3, 12.0, 5.5, 2.1 Hz, 1H), 2.74 (dd, *J* = 15.5, 3.7 Hz, 1H). ^13^C NMR (100 MHz, Chloroform-*d*) δ 172.01, 169.11, 136.43, 133.96, 132.83, 132.78, 128.57, 128.55, 127.91, 127.33, 127.09, 126.88, 126.42, 126.30, 124.11, 122.70, 119.88, 118.42, 111.34, 109.81, 53.03, 52.98, 44.57, 21.84. MS(ESI+) m/z :385.1674[M+H]^+^.

Methyl-2-(benzo [*d*] [1,3] dioxole-5-carbonyl)-2, 3, 4, 9-tetrahydro-*1H*-pyrido [3, 4-b] indole-1-carboxylate (Compound 6i)

Reddish brown solid, yield 73.4%, m.p:165.3~166.7 ℃. ^1^H NMR (400 MHz, Chloroform-*d*) δ 8.45 (s, 1H), 7.74 (d, *J* = 8.0 Hz, 2H), 7.65 (d, *J* = 8.0 Hz, 2H), 7.49 (d, J = 7.8 Hz, 1H), 7.39 (d, *J* = 8.1 Hz, 1H), 7.26 – 7.20 (m, 1H), 7.13 (t, *J* = 7.4 Hz, 1H), 6.25 (s, 1H), 4.00 (dd, *J* = 13.6, 4.6 Hz, 1H), 3.86 (s, 3H), 3.64 (dd, *J* = 18.6, 6.8 Hz, 1H), 2.96 – 2.75 (m, 2H). ^13^C NMR (100 MHz, Chloroform-*d*) δ 170.49, 168.74, 139.06, 136.37, 127.44, 126.18, 125.97, 125.84, 125.80, 125.77, 122.85, 120.00, 118.42, 111.27, 109.66, 53.11, 52.81, 44.43, 21.73. MS(ESI+) m/z :379.1415[M+H]^+^.

Ethyl-2-(4-fluorobenzoyl)-2, 3, 4, 9-tetrahydro-1*H*-pyrido [3, 4-b] indole-1-carboxylate (Compound 7a)

Light yellow solid, yield 53.7%, m.p:199.5~201.7 ℃.^1^H NMR (400 MHz, Chloroform-*d*) δ 8.55 (s, 1H), 7.54 (dd, *J* = 8.5, 5.3 Hz, 2H), 7.49 (d, *J* = 7.9 Hz, 1H), 7.38 (d, *J* = 8.1 Hz, 1H), 7.24 – 7.10 (m, 4H), 6.37 – 5.92 (m, 1H), 4.33 – 4.25 (m, 2H), 4.09 (dd, *J* = 13.9, 4.6 Hz, 1H), 3.63 (ddd, *J* = 13.7, 11.8, 4.0 Hz, 1H), 2.96 – 2.82 (m, 1H), 2.77 (dd, *J* = 15.5, 3.8 Hz, 1H), 1.32 (t, *J* = 7.1 Hz, 3H). ^13^C NMR (100 MHz, Chloroform-*d*) δ 170.98, 168.50, 136.41, 129.36, 126.50, 126.25, 122.66, 119.87, 118.36, 115.89, 115.68, 111.32, 109.63, 62.24, 53.12, 44.61, 21.82, 14.30. MS(ESI+) m/z: 367.2346 [M+H]^+^.

Ethyl-2-(4-methoxybenzoyl)-2, 3, 4, 9-tetrahydro-1H-pyrido [3, 4-b] indole-1-carboxylate (Compound 7b)

White solid, yield 74.8%, m.p:206.9~209.8℃.^1^H NMR (400 MHz, Chloroform-*d*) δ 8.75 (s, 1H), 7.63 – 7.53 (m, 3H), 7.35 (dd, *J* = 7.4, 1.6 Hz, 1H), 7.23 (td, *J* = 7.5, 1.7 Hz, 1H), 7.16 (td, *J* = 7.4, 1.6 Hz, 1H), 7.07 – 7.00 (m, 2H), 6.08 (s, 1H), 4.52 (dq, *J* = 12.5, 8.1 Hz, 1H), 4.26 (dq, *J* = 12.5, 8.1 Hz, 1H), 3.92 (dt, *J* = 12.5, 7.1 Hz, 1H), 3.80 (s, 3H), 3.59 (dt, *J* = 12.6, 7.1 Hz, 1H), 2.88 (dt, *J* = 17.2, 7.1 Hz, 1H), 2.79 (dt, *J* = 17.0, 7.0 Hz, 1H), 1.22 (t, *J* = 8.0 Hz, 3H). ^13^C NMR (100 MHz, Chloroform-*d*) δ 168.29, 167.64, 162.21, 136.77, 132.65, 132.32, 129.31, 127.44, 121.43, 120.06, 119.48, 113.60, 111.23, 109.10, 62.31, 56.79, 55.35, 42.33, 20.10, 13.94.

Ethyl 2-([1, 1’-biphenyl]-4-carbonyl)-2, 3, 4, 9-tetrahydro-1*H*-pyrido [3, 4-b] indole-1 carboxylate (Compound 7c).

White solid, yield 72.5%, m.p.: 201.4~201.8 ℃ .^1^H-NMR (400 MHz, DMSO-d6, TMS, ppm): δ 11.05(s, 1H), 7.82 (d, *J* = 8.2 Hz, 2H), 7.75 (d, *J* = 7.3 Hz,2H), 7.60 (d, *J* = 8.2 Hz, 2H), 7.52 (t, *J* = 7.6 Hz, 2H),7.43 (dt, *J* = 7.3, 6.9 Hz, 3H), 7.12 (t, *J* = 7.7 Hz, 1H),7.01 (t, *J* = 7.4 Hz, 1H), 6.05 (s, 1H), 4.30~4.20 (m,2H), 4.03 (dd, *J* = 13.9, 3.9 Hz, 1H), 3.59 (dd, *J* = 17.8, 7.8 Hz, 1H), 2.95~2.74 (m, 2H), 1.29 (t, *J* =7.1Hz, 3H). 13C NMR (100 MHz, DMSO-*d_6_*): δ 171.17, 169.09, 142.36, 139.77, 137.11,134.82, 129.61, 128.55, 128.18, 127.47, 127.38, 127.19, 126.29, 122.23, 119.38, 118.58, 112.22, 108.93, 62.12, 53.42, 44.66, 21.63, 14.63. MS(ESI+) m/z: 447.1748. [M+Na]^+^

Ethyl-2-(4-iodobenzoyl)-2,3,4,9-tetrahydro-*1H*-pyrido[3,4-b] indole-1-carboxylate (Compound 7d)

White solid, yield 75.9%, m.p:175.6~176.3℃ ^1^H NMR (400 MHz, Chloroform-*d*) δ 8.41 (s, 1H), 7.82 (d, *J* = 8.1 Hz, 2H), 7.49 (d, *J* = 7.8 Hz, 1H), 7.40 (d, *J* = 8.1 Hz, 1H), 7.28 (s, 1H), 7.22 (d, *J* = 7.6 Hz, 1H), 7.13 (t, *J* = 7.5 Hz, 2H), 6.19 (s, 1H), 4.30 (dq, *J* = 7.3, 4.1 Hz, 2H), 4.06 (dd, *J* = 13.7, 5.0 Hz, 1H), 3.66 – 3.57 (m, 1H), 2.94 – 2.84 (m, 1H), 2.77 (dd, *J* = 15.5, 3.9 Hz, 1H), 1.35 (t, *J* = 7.2 Hz,3H).^13^CNMR(100MHz, Chloroform-d) 170.97, 168.39, 137.84, 136.33, 135.00, 128.74, 126.37, 126.24, 122.73, 119.93, 118.39, 111.25, 109.68, 96.42, 62.28, 52.96, 44.50, 21.79, 14.32. MS(ESI+) m/z: 475.0538[M+H]^+^.

Ethyl-2-benzoyl-2, 3, 4, 9-tetrahydro-*1H*-pyrido [3, 4-b] indole-1-carboxylate (Compound 7e)

White powder, yield 72.6%, m.p:196.5~198.6℃.^1^H NMR (500 MHz, Chloroform-d) δ 8.75 (s, 1H), 7.56 (ddd, *J* = 7.3, 3.1, 1.7 Hz, 3H), 7.41 (t, *J* = 7.5 Hz, 2H), 7.38 – 7.27 (m, 2H), 7.23 (td, *J* = 7.5, 1.7 Hz, 1H), 7.16 (td, *J* = 7.4, 1.5 Hz, 1H), 6.01 (s, 1H), 3.79 (dt, *J* = 12.4, 6.9 Hz, 1H), 3.63 (dt, *J* = 12.6, 7.1 Hz, 1H), 3.09 (dq, *J* = 12.5, 8.0 Hz, 1H), 2.91 (dt, *J* = 16.8, 7.1 Hz, 1H), 2.83 (dt, *J* = 16.6, 7.1 Hz, 1H), 2.62 (dq, *J* = 12.4, 8.0 Hz, 1H), 1.20 (t, *J* = 8.0 Hz, 3H). ^13^C NMR (125 MHz) δ 169.43, 168.29, 136.77, 135.85, 133.79, 131.87, 128.73, 127.44, 126.45, 121.43, 120.06, 119.48, 111.23, 107.31, 61.09, 42.33, 34.41, 20.10, 8.52. MS(ESI+) m/z: 371.1397[M+Na]^+^.

Ethyl-2-(4-(trifluoromethyl)benzoyl)-2, 3, 4, 9-tetrahydro-*1H*-pyrido [3, 4-b] indole-1-carboxylate (Compound 7f)

Red powder, yield 79.3%, m.p:210.2~212.6℃.1H NMR (400 MHz, Chloroform-*d*) δ 8.47 (s, 1H), 7.74 (d, *J* = 7.9 Hz, 2H), 7.64 (d, *J* = 8.0 Hz, 2H), 7.49 (d, *J* = 7.9 Hz, 1H), 7.40 (d, *J* = 8.1 Hz, 1H), 7.26 – 7.19 (m, 1H), 7.13 (t, *J* = 7.5 Hz, 1H), 6.24 (s, 1H), 4.30 (ddt, *J* = 9.8, 7.1, 2.9 Hz, 2H), 4.06 – 3.92 (m, 1H), 3.63 (ddd, *J* = 13.5, 11.7, 4.2 Hz, 1H), 2.94 – 2.74 (m, 2H), 1.35 (t, *J* = 7.1 Hz, 3H). 13C NMR (100MHz, Chloroform-d) δ 170.45, 168.29, 139.17, 136.38, 132.23, 131.90, 127.40, 126.92, 126.20, 126.19, 125.81, 125.77, 122.78, 119.96, 118.39, 111.29, 109.58, 62.37, 52.92, 44.45, 21.75, 14.31. MS(ESI+) m/z: 417.1758 [M+H]^+^.

Ethyl-2-(4-chlorobenzoyl)-2,3,4,9-tetrahydro-*1H*-pyrido[3,4-b] indole-1-carboxylate (Compound 7g)

Light brown powder, yield 49.8%, m.p.:232.3~233.5 ℃ ^1^H NMR (400 MHz, Chloroform-*d*) δ 8.75 (s, 1H), 7.77 – 7.70 (m, 2H), 7.65 – 7.58 (m, 2H), 7.56 (dd, *J* = 7.4, 1.7 Hz, 1H), 7.35 (dd, *J* = 7.4, 1.6 Hz, 1H), 7.23 (td, *J* = 7.5, 1.7 Hz, 1H), 7.16 (td, *J* = 7.4, 1.6 Hz, 1H), 6.03 (s, 1H), 3.95 (dt, *J* = 12.5, 7.0 Hz, 1H), 3.81 (s, 3H), 3.61 (dt, *J* = 12.5, 7.0 Hz, 1H), 2.92 (dt, *J* = 17.0, 7.1 Hz, 1H), 2.82 (dt, *J* = 17.0, 7.0 Hz, 1H). ^13^C NMR (125 MHz, Chloroform-*d*) δ 169.54, 168.29, 136.77, 135.63, 132.65, 128.98, 128.78, 127.44, 121.43, 120.06, 119.48, 111.23, 109.10, 56.30, 52.88, 42.33, 20.10.

Ethyl-2-(2-naphthoyl)-2, 3, 4, 9-tetrahydro-1H-pyrido [3, 4-b] indole-1-carboxylate (Compound 7h)

Light yellow solid, yield 73.6%, m.p.: 209.6~211.5℃ ^1^H NMR (400 MHz, Chloroform-*d*) δ 8.55 (s, 1H), 8.21 (t, *J* = 1.4 Hz, 1H), 7.99 (dd, *J* = 7.5, 1.6 Hz, 1H), 7.89 (tt, *J* = 8.9, 1.5 Hz, 2H), 7.62 (td, *J* = 7.5, 1.6 Hz, 2H), 7.59 – 7.49 (m, 2H), 7.35 (dd, *J* = 7.5, 1.5 Hz, 1H), 7.23 (td, *J* = 7.5, 1.7 Hz, 1H), 7.16 (td, *J* = 7.4, 1.5 Hz, 1H), 6.05 (s, 1H), 4.07 (dt, *J* = 12.4, 7.1 Hz, 1H), 3.60 (dt, *J* = 12.4, 7.0 Hz, 1H), 3.47 (dq, *J* = 12.4, 8.0 Hz, 1H), 3.05 – 2.87 (m, 2H), 2.82 (dt, *J* = 17.0, 7.1 Hz, 1H), 1.25 (t, *J* = 8.0 Hz, 3H). ^13^C NMR (100 MHz, DMSO-*d_6_*) δ 204.43, 169.58, 136.77, 135.85, 133.50, 133.40, 132.83, 128.61, 127.85, 127.57, 127.44, 127.00, 126.11, 123.73, 121.43, 120.06, 119.48, 111.23, 107.31, 61.09, 42.33, 34.41, 20.10, 8.52. MS(ESI+) m/z: 399.1721[M+H]^+^.

Ethyl-2-(benzo [*d*] [1,3] dioxole-5-carbonyl)-2, 3, 4, 9-tetrahydro-*1H*-pyrido [3, 4-b] indole-1-carboxylate (Compound 7i)

Light brown powder, yield 45.3%, m.p:202.5~204.6℃.^1^H NMR (400 MHz, Chloroform-*d*) δ 8.47 (s, 1H), 7.49 (d, *J* = 7.8 Hz, 1H), 7.39 (d, *J* = 8.1 Hz, 1H), 7.22 (dd, *J* = 15.2, 7.8 Hz, 1H), 7.12 (t, *J* = 7.5 Hz, 1H), 7.09 – 6.99 (m, 2H), 6.88 (d, *J* = 7.8 Hz, 1H), 6.16 (s, 1H), 6.03 (s, 2H), 4.29 (qt, *J* = 7.1, 3.1 Hz, 2H), 4.22 – 4.10 (m, 1H), 3.62 (td, *J* = 13.6, 12.8, 4.1 Hz, 1H), 3.02 – 2.74 (m, 2H), 1.33 (t, *J* = 7.1 Hz, 3H). ^13^C NMR (100 MHz, Chloroform-*d*) δ 171.30, 168.62, 149.22, 147.76, 136.36, 129.18, 126.69, 126.30, 122.61, 121.64, 119.84, 118.36, 111.26, 109.77, 108.38, 108.01, 101.54, 62.16, 53.20, 44.67, 21.85, 14.31. MS(ESI+) m/z :415.1295[M+Na]^+^.
